# 4-{[4-(Di­methyl­amino)­benzyl­idene]amino}­benzene­sulfonamide

**DOI:** 10.1107/S1600536814012136

**Published:** 2014-05-31

**Authors:** Mustafa Durgun, Hasan Türkmen, Tuncay Tunç, Tuncer Hökelek

**Affiliations:** a Department of Chemistry, Harran University, 63300 Şanlıurfa, Turkey; b Science Education Department, Aksaray University, 68100 Aksaray, Turkey; c Department of Physics, Hacettepe University, 06800 Beytepe, Ankara, Turkey

## Abstract

The title Schiff base compound, C_15_H_17_N_3_O_2_S, is non-planar with a dihedral angle of 69.88 (4)° between the planes of the benzene rings. In the crystal, pairs of N—H⋯N hydrogen bonds, between the sulfonamide nitro­gen-H atom and the azomethine N atom, link the mol­ecules into inversion dimers, forming *R*_2_^2^(16) ring motifs. These dimers are linked by N—H⋯O hydrogen bonds, between the sulfonamide nitro­gen-H atom and one sulfonamide O atom, forming sheets lying parallel to (100). Within the sheets there are weak parallel slipped π–π inter­actions involving inversion-related benzene­sulfonamide rings [centroid–centroid distance = 3.8800 (9) Å; normal distance = 3.4796 (6) Å; slippage = 1.717 Å].

## Related literature   

For the biological and physical properties of sulfonamides and their derivatives and for their pharmacological applications, see: Chohan & Shad (2012 ▶); Domagk (1935 ▶); Khalil *et al.* (2009 ▶); Sharaby (2007 ▶); Lin *et al.* (2008 ▶); Maren (1967 ▶); Mohamed *et al.* (2013 ▶); Saluja *et al.* (2014 ▶); Supuran *et al.* (1996 ▶); Türkmen *et al.* (2005 ▶). For related structures, see: Idemudia *et al.* (2012 ▶); Loughrey *et al.* (2009 ▶). For bond-length data, see: Allen *et al.* (1987 ▶). For graph-set analysis, see: Bernstein *et al.* (1995 ▶).
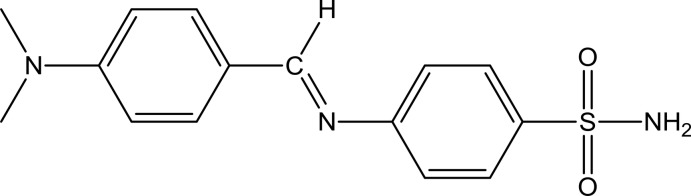


## Experimental   

### 

#### Crystal data  

C_15_H_17_N_3_O_2_S
*M* *_r_* = 303.38
Monoclinic, 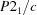

*a* = 16.8982 (5) Å
*b* = 9.0273 (3) Å
*c* = 9.8405 (3) Å
β = 101.552 (3)°
*V* = 1470.71 (8) Å^3^
*Z* = 4
Mo *K*α radiation
μ = 0.23 mm^−1^
*T* = 296 K
0.35 × 0.22 × 0.15 mm

#### Data collection  

Bruker SMART BREEZE CCD diffractometer
Absorption correction: multi-scan (*SADABS*; Bruker, 2012 ▶) *T*_min_ = 0.924, *T*_max_ = 0.987
19398 measured reflections
3644 independent reflections
3133 reflections with *I* > 2σ(*I*)
*R*_int_ = 0.025

#### Refinement  

*R*[*F* ^2^ > 2σ(*F* ^2^)] = 0.040
*wR*(*F* ^2^) = 0.116
*S* = 1.08
3644 reflections
204 parameters
H atoms treated by a mixture of independent and constrained refinement
Δρ_max_ = 0.37 e Å^−3^
Δρ_min_ = −0.29 e Å^−3^

### 

Data collection: *APEX2* (Bruker, 2012 ▶); cell refinement: *SAINT* (Bruker, 2012 ▶); data reduction: *SAINT*; program(s) used to solve structure: *SHELXS97* (Sheldrick, 2008 ▶); program(s) used to refine structure: *SHELXL97* (Sheldrick, 2008 ▶); molecular graphics: *ORTEP-3 for Windows* (Farrugia, 2012 ▶); software used to prepare material for publication: *WinGX* (Farrugia, 2012 ▶) and *PLATON* (Spek, 2009 ▶).

## Supplementary Material

Crystal structure: contains datablock(s) I, global. DOI: 10.1107/S1600536814012136/su2738sup1.cif

Structure factors: contains datablock(s) I. DOI: 10.1107/S1600536814012136/su2738Isup2.hkl

Supporting information file. DOI: 10.1107/S1600536814012136/su2738Isup3.cml

CCDC reference: 1005250

Additional supporting information:  crystallographic information; 3D view; checkCIF report

## Figures and Tables

**Table 1:** Hydrogen-bond geometry (Å, °)

*D*—H⋯*A*	*D*—H	H⋯*A*	*D*⋯*A*	*D*—H⋯*A*
N3—H31⋯N2^i^	0.80 (3)	2.18 (3)	2.981 (2)	177 (2)
N3—H32⋯O2^ii^	0.832 (19)	2.494 (19)	3.321 (2)	174 (2)

Symmetry codes: (i) 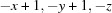; (ii) 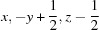.

**Fig. 1.**
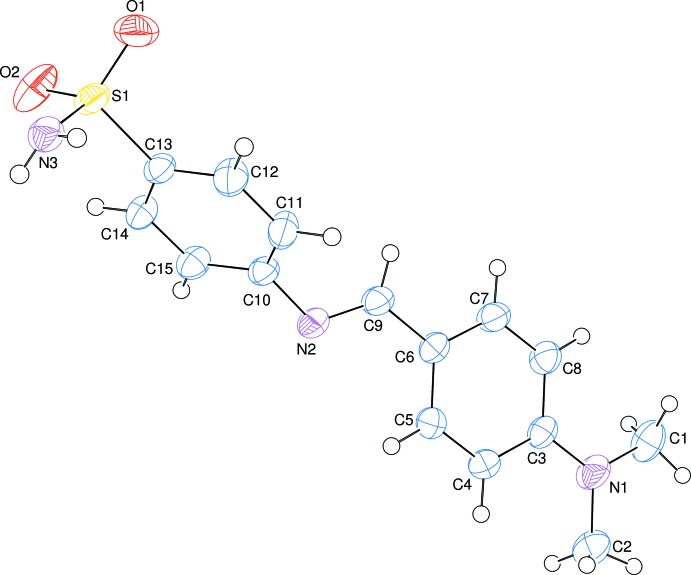
The molecular structure of the title molecule, with atom labelling. Displacement ellipsoids are drawn at the 50% probability level.

**Fig. 2.**
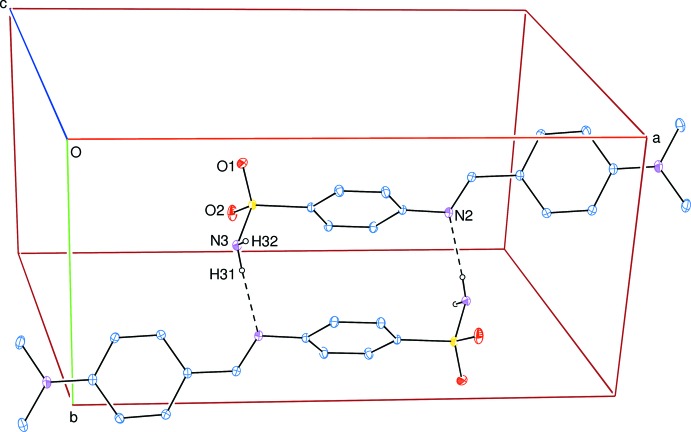
A partial view along the c axis of the crystal packing of the title compound. The N—H···N hydrogen bonds, linking the molecules into inversion dimes and forming R22(16) ring motifs, are shown as dashed lines (see Table 1 for details; C bound H atoms have been omitted for clarity).

## supplementary crystallographic information

### 1. Comment

Many Schiff bases are prepared by condensation reactions of a sulfonamide with a substituted benzaldehyde derivative. Such compounds contain both azomethine (-HC═N-) and sulfonamide (-SO_2_NH_2_) groups. Sulfonamide derivatives are very important because of their varied structures and biological activities (Domagk, 1935). This type of derivative displays interesting enzymatic inhibition towards the carbonic anhydrase (CA) isozymes CA I, II, IV, IX and XII (Supuran *et al.*, 1996; Türkmen *et al.*, 2005; Saluja *et al.*, 2014) and the *cyclo*-oxygenase (COX) enzymes COX-1 and COX-2 (Lin *et al.*, 2008) are still widely used as antimicrobial drugs, antithyroid agents, antibacterial, antifungal, antitumor, antibiotics, acid-base indicator, potential anticancer agents (Maren, 1967; Khalil *et al.*, 2009; Sharaby, 2007; Mohamed *et al.*, 2013; Chohan & Shad, 2012). The title compound was synthesized and its crystal structure is reported on herein.

In the molecule of the title compound (Fig. 1) the bond lengths are within normal ranges (Allen *et al.*, 1987). The azomethine (-HC═N-) group is rotated out of the plane of the dimethylamino benzaldehyde and benzenesulfonamide benzene rings with torsion angles C5–C6–C9–N2 = -15.4 (2)° and C11–C10–N2–c9 = -53.6 (2)°. The two benzene rings *A* (C3—C8) and *B* (C10—C15) are oriented at a dihedral angle of 69.88 (4)°. Atoms N1, N2, C1, C2, C9 and C10 atoms are displaced from the plane of ring A by 0.034 (2), -0.302 (1), -0.045 (2), 0.106 (2), -0.018 (2) and -0.146 (2) Å, respectively, while atoms S1 and N2 are displaced from ring B by -0.0181 (4) and 0.026 (1) Å, respectively.

In the crystal, pairs of N—H···N hydrogen bonds link the molecules into inversion dimers forming R^2^_2_(16) ring motifs (Table 1 and Fig. 2; Bernstein *et al.*, 1995). These dimers are linked by N—H···O hydrogen bonds (Table 1) forming sheets lying parallel to (100). A π···π contact between inversion related the benzenesulfonamide benzene rings in the sheet, Cg2—Cg2^i^ [symmetry code: (i) - *x+1*, - *y+ 2*, - *z;* where Cg2 is the centroid of ring *B*] may further stabilize the structure, with a centroid-centroid distance of 3.8800 (9) Å.

### 2. Experimental

The title compound was synthesized according to the literature method with some modifications (Khalil *et al.*, 2009; Sharaby, 2007; Lin *et al.*, 2008; Supuran *et al.*, 1996; Mohamed *et al.*, 2013). Sulfonamide (0.172 g, 1.0 mmol) in absolute ethanol (20 ml) was added to 4-(dimethylamino)benzaldehyde (0.149 g, 1.0 mmol) in absolute ethanol (20 ml), and 2 drops of formic acid were added as catalyst. The mixture was refluxed for 3-4 h, followed by cooling to room temperature. The resulting crystals were filtered in vacuum (yield: 85%). Crystals suitable for X-ray analysis were grown by slow evaporation of a methanol/ethanol/chloroform (3:1:1) solution, giving yellow prismatic crystals.

### 3. Refinement

Atoms H31, H32 (for NH_2_) and H9 (for CH) were located in a difference Fourier map and freely refined. The remaining C-bound H-atoms were positioned geometrically with C—H = 0.93 and 0.96 Å for aromatic and methyl H-atoms, respectively, and constrained to ride on their parent atoms, with *U*_iso_(H) = 1.5*U*_eq_(C-methyl) and = 1.2U_eq_(C) for other H-atoms.

### Crystal data

**Table:**

C_15_H_17_N_3_O_2_S	*F*(000) = 640
*M**_r_* = 303.38	*D*_x_ = 1.370 Mg m^−^^3^
Monoclinic, *P*2_1_/*c*	Mo *K*α radiation, λ = 0.71073 Å
Hall symbol: -P 2ybc	Cell parameters from 9899 reflections
*a* = 16.8982 (5) Å	θ = 2.3–28.3°
*b* = 9.0273 (3) Å	µ = 0.23 mm^−^^1^
*c* = 9.8405 (3) Å	*T* = 296 K
β = 101.552 (3)°	Prism, yellow
*V* = 1470.71 (8) Å^3^	0.35 × 0.22 × 0.15 mm
*Z* = 4	

### Data collection

**Table:**

Bruker SMART BREEZE CCD diffractometer	3644 independent reflections
Radiation source: fine-focus sealed tube	3133 reflections with *I* > 2σ(*I*)
Graphite monochromator	*R*_int_ = 0.025
φ and ω scans	θ_max_ = 28.3°, θ_min_ = 1.2°
Absorption correction: multi-scan (*SADABS*; Bruker, 2012)	*h* = −22→22
*T*_min_ = 0.924, *T*_max_ = 0.987	*k* = −7→12
19398 measured reflections	*l* = −11→13

### Refinement

**Table:**

Refinement on *F*^2^	Primary atom site location: structure-invariant direct methods
Least-squares matrix: full	Secondary atom site location: difference Fourier map
*R*[*F*^2^ > 2σ(*F*^2^)] = 0.040	Hydrogen site location: inferred from neighbouring sites
*wR*(*F*^2^) = 0.116	H atoms treated by a mixture of independent and constrained refinement
*S* = 1.08	*w* = 1/[σ^2^(*F*_o_^2^) + (0.0572*P*)^2^ + 0.485*P*] where *P* = (*F*_o_^2^ + 2*F*_c_^2^)/3
3644 reflections	(Δ/σ)_max_ = 0.001
204 parameters	Δρ_max_ = 0.37 e Å^−^^3^
0 restraints	Δρ_min_ = −0.29 e Å^−^^3^

### Special details

**Table:**

Geometry. All esds (except the esd in the dihedral angle between two l.s. planes) are estimated using the full covariance matrix. The cell esds are taken into account individually in the estimation of esds in distances, angles and torsion angles; correlations between esds in cell parameters are only used when they are defined by crystal symmetry. An approximate (isotropic) treatment of cell esds is used for estimating esds involving l.s. planes.
Refinement. Refinement of F^2^ against ALL reflections. The weighted R-factor wR and goodness of fit S are based on F^2^, conventional R-factors R are based on F, with F set to zero for negative F^2^. The threshold expression of F^2^ > 2sigma(F^2^) is used only for calculating R-factors(gt) etc. and is not relevant to the choice of reflections for refinement. R-factors based on F^2^ are statistically about twice as large as those based on F, and R- factors based on ALL data will be even larger.

### Fractional atomic coordinates and isotropic or equivalent isotropic displacement parameters (Å^2^)

**Table:**

	*x*	*y*	*z*	*U*_iso_*/*U*_eq_	
S1	0.329465 (19)	0.28756 (4)	0.08578 (4)	0.03754 (13)	
O1	0.30989 (7)	0.13297 (14)	0.08516 (15)	0.0608 (4)	
O2	0.31107 (7)	0.37779 (18)	0.19404 (13)	0.0613 (4)	
N1	1.05511 (8)	0.16049 (17)	0.10070 (15)	0.0479 (3)	
N2	0.68218 (7)	0.32679 (14)	0.09636 (13)	0.0364 (3)	
N3	0.28201 (7)	0.35422 (17)	−0.05859 (14)	0.0385 (3)	
H31	0.2899 (12)	0.441 (3)	−0.069 (2)	0.058 (6)*	
H32	0.2864 (13)	0.300 (2)	−0.125 (2)	0.059 (6)*	
C1	1.10626 (9)	0.0462 (2)	0.17517 (18)	0.0516 (4)	
H1A	1.1559	0.0414	0.1419	0.077*	
H1B	1.0791	−0.0476	0.1606	0.077*	
H1C	1.1178	0.0690	0.2724	0.077*	
C2	1.09209 (11)	0.2638 (2)	0.0203 (2)	0.0611 (5)	
H2A	1.1451	0.2294	0.0148	0.092*	
H2B	1.0961	0.3593	0.0640	0.092*	
H2C	1.0597	0.2714	−0.0715	0.092*	
C3	0.97582 (8)	0.17399 (17)	0.10927 (15)	0.0350 (3)	
C4	0.92808 (9)	0.29053 (16)	0.04153 (18)	0.0410 (3)	
H4	0.9514	0.3603	−0.0079	0.049*	
C5	0.84765 (9)	0.30314 (16)	0.04715 (17)	0.0394 (3)	
H5	0.8178	0.3817	0.0021	0.047*	
C6	0.81000 (8)	0.20063 (16)	0.11890 (15)	0.0347 (3)	
C7	0.85645 (9)	0.08345 (18)	0.18336 (16)	0.0405 (3)	
H7	0.8321	0.0119	0.2292	0.049*	
C8	0.93761 (9)	0.07021 (18)	0.18125 (15)	0.0405 (3)	
H8	0.9672	−0.0079	0.2277	0.049*	
C9	0.72524 (8)	0.21083 (16)	0.12705 (16)	0.0363 (3)	
H9	0.7038 (10)	0.128 (2)	0.1561 (17)	0.041 (4)*	
C10	0.59860 (8)	0.31581 (15)	0.09662 (14)	0.0321 (3)	
C11	0.55164 (9)	0.20677 (16)	0.01965 (17)	0.0408 (3)	
H11	0.5755	0.1385	−0.0305	0.049*	
C12	0.46948 (9)	0.19932 (17)	0.01722 (18)	0.0416 (3)	
H12	0.4382	0.1260	−0.0340	0.050*	
C13	0.43431 (8)	0.30106 (15)	0.09101 (14)	0.0321 (3)	
C14	0.47978 (8)	0.41333 (17)	0.16501 (15)	0.0363 (3)	
H14	0.4554	0.4829	0.2129	0.044*	
C15	0.56207 (8)	0.42089 (17)	0.16691 (15)	0.0372 (3)	
H15	0.5928	0.4965	0.2154	0.045*	

### Atomic displacement parameters (Å^2^)

**Table:**

	*U*^11^	*U*^22^	*U*^33^	*U*^12^	*U*^13^	*U*^23^
S1	0.02420 (17)	0.0491 (2)	0.0407 (2)	0.00001 (13)	0.00964 (14)	0.00972 (15)
O1	0.0358 (6)	0.0526 (7)	0.0930 (10)	−0.0060 (5)	0.0103 (6)	0.0303 (7)
O2	0.0387 (6)	0.1018 (11)	0.0477 (7)	0.0046 (7)	0.0191 (5)	−0.0089 (7)
N1	0.0281 (6)	0.0592 (8)	0.0583 (8)	0.0037 (6)	0.0131 (6)	0.0048 (7)
N2	0.0254 (5)	0.0400 (6)	0.0449 (7)	−0.0016 (5)	0.0095 (5)	0.0025 (5)
N3	0.0281 (6)	0.0441 (7)	0.0425 (7)	0.0011 (5)	0.0055 (5)	0.0067 (6)
C1	0.0308 (7)	0.0734 (12)	0.0487 (9)	0.0107 (7)	0.0030 (6)	−0.0042 (8)
C2	0.0372 (8)	0.0600 (11)	0.0927 (15)	−0.0065 (8)	0.0292 (9)	0.0010 (10)
C3	0.0274 (6)	0.0421 (7)	0.0356 (7)	−0.0013 (5)	0.0069 (5)	−0.0062 (6)
C4	0.0335 (7)	0.0382 (8)	0.0544 (9)	−0.0018 (6)	0.0164 (6)	0.0037 (6)
C5	0.0324 (7)	0.0362 (7)	0.0508 (9)	0.0038 (5)	0.0116 (6)	0.0060 (6)
C6	0.0259 (6)	0.0396 (7)	0.0390 (7)	−0.0001 (5)	0.0073 (5)	−0.0001 (6)
C7	0.0330 (7)	0.0456 (8)	0.0444 (8)	0.0016 (6)	0.0116 (6)	0.0103 (6)
C8	0.0322 (7)	0.0481 (8)	0.0410 (8)	0.0073 (6)	0.0068 (6)	0.0073 (6)
C9	0.0281 (6)	0.0391 (8)	0.0426 (8)	−0.0020 (5)	0.0092 (6)	0.0047 (6)
C10	0.0245 (6)	0.0363 (7)	0.0357 (7)	−0.0003 (5)	0.0066 (5)	0.0051 (5)
C11	0.0306 (7)	0.0387 (8)	0.0541 (9)	0.0017 (5)	0.0110 (6)	−0.0104 (6)
C12	0.0307 (7)	0.0386 (8)	0.0543 (9)	−0.0042 (5)	0.0055 (6)	−0.0099 (6)
C13	0.0241 (6)	0.0385 (7)	0.0340 (7)	0.0007 (5)	0.0067 (5)	0.0051 (5)
C14	0.0319 (7)	0.0419 (7)	0.0368 (7)	0.0011 (6)	0.0111 (5)	−0.0053 (6)
C15	0.0309 (6)	0.0419 (8)	0.0385 (7)	−0.0056 (6)	0.0068 (5)	−0.0073 (6)

### Geometric parameters (Å, º)

**Table:**

S1—O1	1.4340 (13)	C4—H4	0.9300
S1—O2	1.4239 (13)	C5—H5	0.9300
S1—N3	1.6023 (13)	C6—C5	1.393 (2)
S1—C13	1.7664 (13)	C6—C7	1.392 (2)
N1—C1	1.447 (2)	C6—C9	1.4534 (18)
N1—C2	1.444 (2)	C7—C8	1.3809 (19)
N2—C9	1.2762 (19)	C7—H7	0.9300
N2—C10	1.4163 (16)	C8—H8	0.9300
N3—H31	0.81 (2)	C9—H9	0.904 (18)
N3—H32	0.83 (2)	C10—C15	1.389 (2)
C1—H1A	0.9600	C10—C11	1.390 (2)
C1—H1B	0.9600	C11—H11	0.9300
C1—H1C	0.9600	C12—C11	1.385 (2)
C2—H2A	0.9600	C12—H12	0.9300
C2—H2B	0.9600	C13—C12	1.377 (2)
C2—H2C	0.9600	C13—C14	1.3869 (19)
C3—N1	1.3647 (18)	C14—C15	1.3887 (18)
C3—C4	1.409 (2)	C14—H14	0.9300
C3—C8	1.407 (2)	C15—H15	0.9300
C4—C5	1.376 (2)		
			
O2—S1—O1	118.37 (9)	C4—C5—H5	119.3
O1—S1—N3	106.72 (8)	C6—C5—H5	119.3
O1—S1—C13	107.24 (7)	C5—C6—C9	122.76 (13)
O2—S1—N3	107.64 (8)	C7—C6—C5	117.57 (13)
O2—S1—C13	107.86 (7)	C7—C6—C9	119.67 (13)
N3—S1—C13	108.72 (7)	C6—C7—H7	119.0
C2—N1—C1	117.24 (14)	C8—C7—C6	122.00 (14)
C3—N1—C1	121.77 (14)	C8—C7—H7	119.0
C3—N1—C2	120.95 (14)	C3—C8—H8	119.8
C9—N2—C10	117.63 (12)	C7—C8—C3	120.48 (14)
S1—N3—H31	114.2 (14)	C7—C8—H8	119.8
S1—N3—H32	111.8 (14)	N2—C9—C6	124.09 (13)
H32—N3—H31	116 (2)	N2—C9—H9	120.7 (11)
N1—C1—H1A	109.5	C6—C9—H9	115.2 (11)
N1—C1—H1B	109.5	C11—C10—N2	120.62 (13)
N1—C1—H1C	109.5	C15—C10—N2	119.72 (12)
H1A—C1—H1B	109.5	C15—C10—C11	119.52 (12)
H1A—C1—H1C	109.5	C10—C11—H11	119.8
H1B—C1—H1C	109.5	C12—C11—C10	120.31 (13)
N1—C2—H2A	109.5	C12—C11—H11	119.8
N1—C2—H2B	109.5	C11—C12—H12	120.2
N1—C2—H2C	109.5	C13—C12—C11	119.69 (13)
H2A—C2—H2B	109.5	C13—C12—H12	120.2
H2A—C2—H2C	109.5	C12—C13—S1	118.35 (11)
H2B—C2—H2C	109.5	C12—C13—C14	120.80 (12)
N1—C3—C4	120.90 (14)	C14—C13—S1	120.84 (11)
N1—C3—C8	121.80 (14)	C13—C14—C15	119.40 (13)
C8—C3—C4	117.27 (12)	C13—C14—H14	120.3
C3—C4—H4	119.4	C15—C14—H14	120.3
C5—C4—C3	121.27 (13)	C10—C15—H15	119.9
C5—C4—H4	119.4	C14—C15—C10	120.22 (13)
C4—C5—C6	121.37 (14)	C14—C15—H15	119.9
			
O1—S1—C13—C12	37.32 (14)	C7—C6—C5—C4	−0.9 (2)
O1—S1—C13—C14	−144.13 (13)	C9—C6—C5—C4	179.91 (14)
O2—S1—C13—C12	165.83 (13)	C5—C6—C7—C8	2.1 (2)
O2—S1—C13—C14	−15.62 (14)	C9—C6—C7—C8	−178.63 (14)
N3—S1—C13—C12	−77.72 (14)	C5—C6—C9—N2	−15.4 (2)
N3—S1—C13—C14	100.83 (13)	C7—C6—C9—N2	165.43 (16)
C10—N2—C9—C6	174.08 (13)	C6—C7—C8—C3	−1.9 (2)
C9—N2—C10—C11	−53.6 (2)	N2—C10—C11—C12	−178.14 (14)
C9—N2—C10—C15	130.68 (15)	C15—C10—C11—C12	−2.5 (2)
C4—C3—N1—C1	176.79 (15)	N2—C10—C15—C14	178.40 (13)
C4—C3—N1—C2	−1.0 (2)	C11—C10—C15—C14	2.7 (2)
C8—C3—N1—C1	−5.5 (2)	C13—C12—C11—C10	0.3 (2)
C8—C3—N1—C2	176.71 (16)	S1—C13—C12—C11	−179.80 (12)
N1—C3—C4—C5	178.58 (15)	C14—C13—C12—C11	1.7 (2)
C8—C3—C4—C5	0.8 (2)	S1—C13—C14—C15	−179.94 (11)
N1—C3—C8—C7	−177.33 (15)	C12—C13—C14—C15	−1.4 (2)
C4—C3—C8—C7	0.4 (2)	C13—C14—C15—C10	−0.8 (2)
C3—C4—C5—C6	−0.6 (2)		

### Hydrogen-bond geometry (Å, º)

**Table:**

*D*—H···*A*	*D*—H	H···*A*	*D*···*A*	*D*—H···*A*
N3—H31···N2^i^	0.80 (3)	2.18 (3)	2.981 (2)	177 (2)
N3—H32···O2^ii^	0.832 (19)	2.494 (19)	3.321 (2)	174 (2)

Symmetry codes: (i) −*x*+1, −*y*+1, −*z*; (ii) *x*, −*y*+1/2, *z*−1/2.
